# Supplementary material for: Analysis of PARP inhibitor toxicity by multidimensional fluorescence microscopy reveals mechanisms of sensitivity and resistance
Source: Nat Commun. 2018 Jul 11;9:2678. doi: 10.1038/s41467-018-05031-9 (PMC6041334; doi:10.1038/s41467-018-05031-9)
Supplement: Supplementary file 1 — Supplementary Information [file 41467_2018_5031_MOESM1_ESM.pdf]

Analysis of PARP inhibitor toxicity

by multidimensional fluorescence microscopy

reveals mechanisms of sensitivity and resistance

Michelena et al.

Supplementary Information

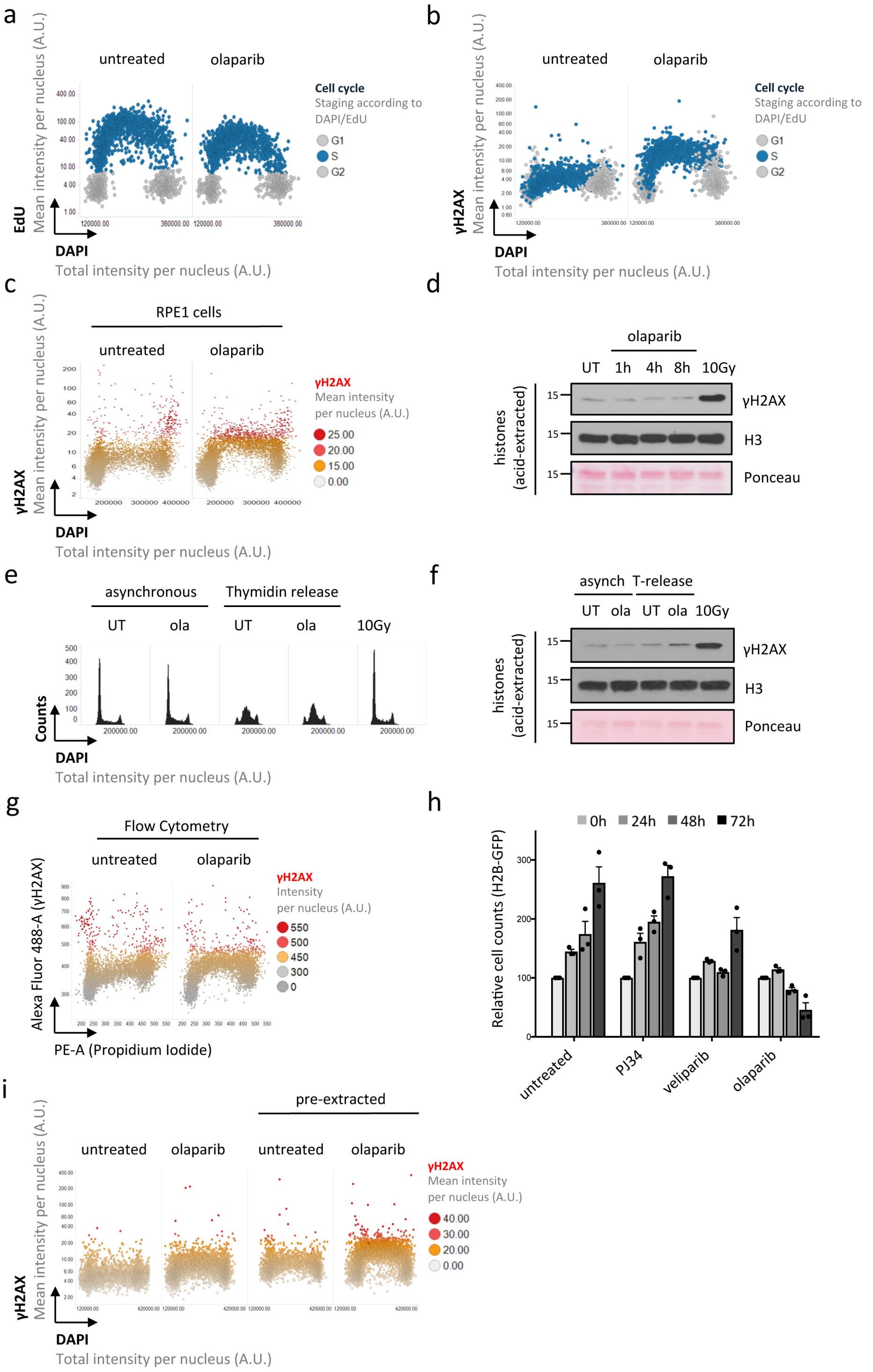

Supplementary Figure 1: Quantitative analysis of PARP inhibitor toxicity by multidimensional fluorescence microscopy. (a) U-2 OS cells were treated with 10 µM olaparib for 4 h, pulsed with EdU for the last 20 min, and stained for EdU, γH2AX and DAPI. Two-dimensional cell cycle staging was performed based on the DAPI/EdU profile. **(b)** For the same cell populations in (a) cell cycle resolved γH2AX formation, color-coded for EdU-positive S-phase cells, is shown. **(c)** RPE1 cells were treated for 4 h with 10 µM olaparib as indicated and cell cycle resolved γH2AX formation is shown. **(d)** U-2 OS cells were treated 10 µM olaparib or irradiated as indicated and histones were purified by acid extraction and probed for γH2AX and total H3. **(e)** U-2 OS cells were synchronized with thymidine for 20 hours and then released in fresh medium in the presence or absence of olaparib (10 µM) for 6 hours. One-dimensional cell cycle staging was performed based on DAPI staining. UT, untreated; ola, olaparib. (**f**) Cells were treated as in (e) and histones were purified by acid extraction and probed for γH2AX and total H3. UT, untreated; ola, olaparib; asynch, asynchronous; T-release, thymidine release. **(g)** U-2 OS cells were treated with olaparib (10 µM) for 4 h, ethanol-fixed, stained with γH2AX and propidium iodide and analyzed by flow cytometry. (**h**) H2B-GFP cells were left untreated or exposed to different PARPi (10 µM) and automated high-content time-lapse microscopy was performed for up to 72 h. Cells were counted based on the H2B-GFP signal. Data are presented as fold-change (mean ± s.d.) relative to the 0 h time point (n=3). (**i**) U-2 OS cells were treated with olaparib (10 µM) for 4 h, fixed or pre-extracted on ice in 0.2% Triton X-100 for 2 min and stained for γH2AX and DAPI. Cell cycle resolved γH2AX formation is shown. Color codes as defined in the figure panels.

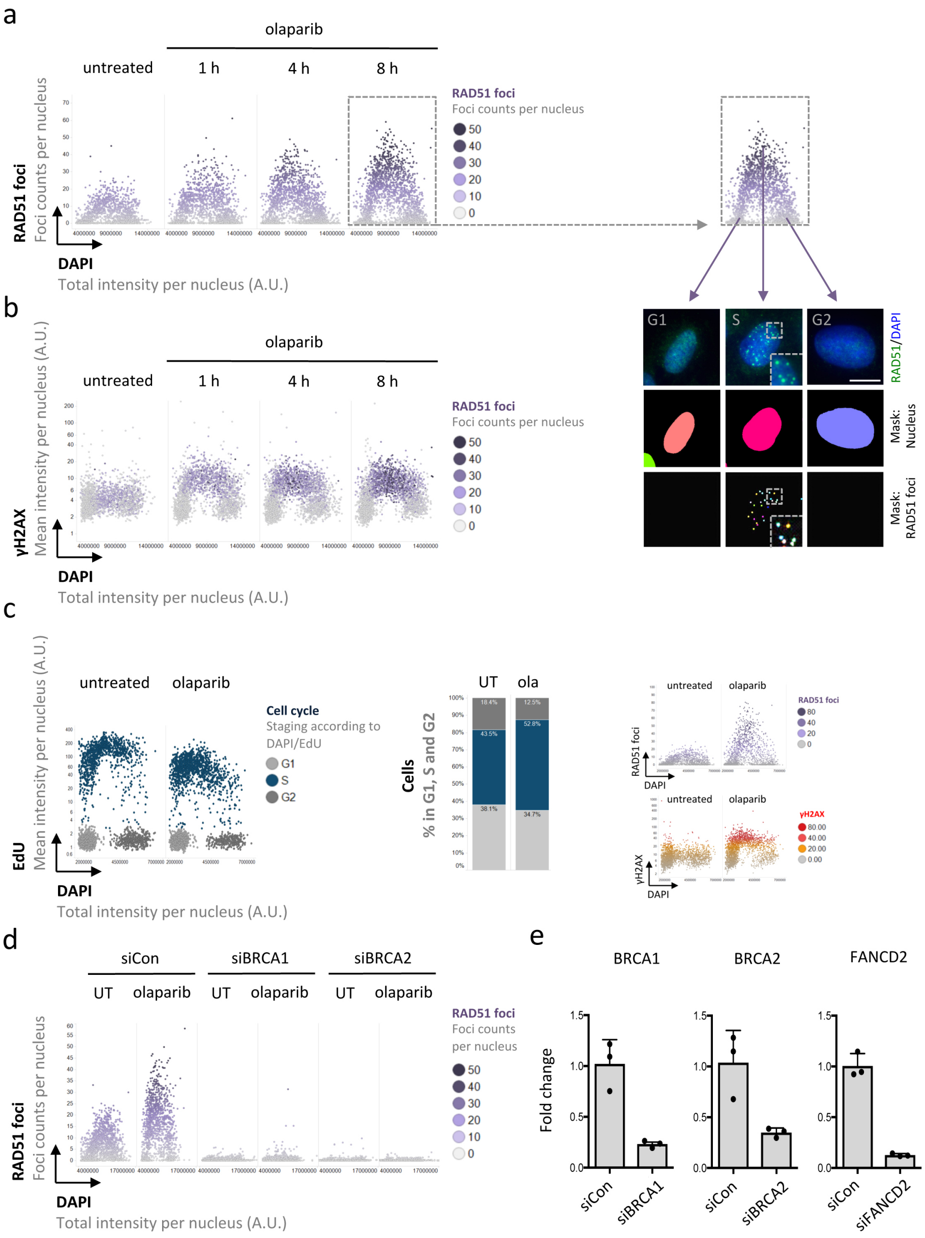

Supplementary Figure 2: Cell cycle resolved quantification of RAD51 accumulation in sub-nuclear foci as measurement of PARPi-induced activation of the HR pathway. **(a)** Cells were treated for the indicated time periods with 10 µM olaparib and stained for RAD51, γH2AX and DNA content. RAD51 foci were quantified in large cohorts of asynchronously growing cells and RAD51 foci numbers per cell are depicted as a function of cell cycle progression. On the right, the masks for nuclei and RAD51 foci detection are shown. **(b)** For the same cell populations cell cycle resolved γH2AX profiles color-coded for the number of RAD51 foci per nucleus are shown. **(c)** U-2 OS cells were treated with 10 µM olaparib for 8 h, pulsed with EdU for the last 20 min, and stained for EdU, γH2AX, RAD51 and DAPI. Two-dimensional cell cycle staging was performed based on the DAPI/EdU profile. Bar charts depict percentages of cells in G1, S and G2. For the same cell populations γH2AX profiles and RAD51 foci counts as a function of cell cycle progression are shown on the right. UT, untreated; ola, olaparib. **(d)** Cells were transfected with negative control siRNA or siRNAs against BRCA1 or BRCA2, treated with 10 µM olaparib for 8 h and stained for RAD51, γH2AX and DNA content. RAD51 foci per nucleus were quantified in large cohorts of asynchronously growing cells and are depicted as a function of cell cycle progression. UT, untreated. (**e**) qPCR analysis confirmed BRCA1, BRCA2 and FANCD2 down-regulation in siRNA-treated samples. Data are presented as fold-change (mean ± s.d.) relative to siCon-transfected samples. Color code as defined in the figure panels.

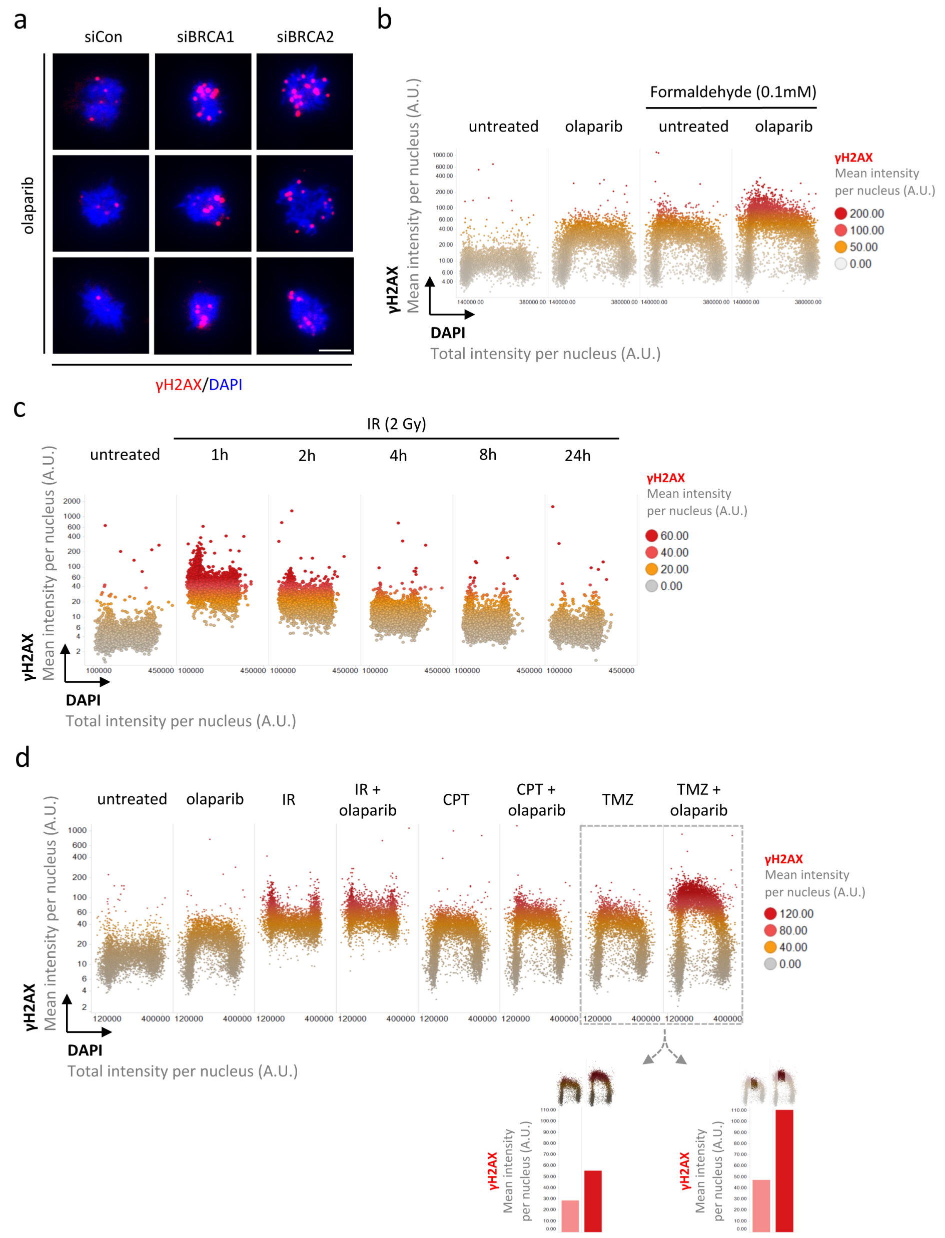

Supplementary Figure 3: Different modes of synergism between PARPi and other genotoxic agents revealed by quantitative image-based cytometry. (a) Cells were transfected with negative control siRNA or siRNAs against BRCA1 or BRCA2, treated with 10 µM olaparib for 8 h, and stained for γH2AX and DNA content. Image-based cell cycle staging identified mitotic cells and individual cell images for each condition are shown. **(b)** Cells were treated with formaldehyde and olaparib (10 µM) for 4 h as indicated and cell cycle resolved γH2AX profiles are shown. **(c)** Cells were treated with IR and allowed to recover for the indicated time-points. Cell cycle resolved γH2AX profiles are shown. **(d)** Cells were treated with single drugs or drug combinations (olaparib 10 µM, IR (ionizing radiation) 2 Gy, CPT (camptothecin) 50 nM, TMZ (temozolomide) 1 mM) for 4 h and cell cycle resolved γH2AX profiles are shown. Below, bar charts depict γH2AX levels as cell population averages from the TMZ- and TMZ+olaparib-treated samples either focused on the whole cell population (bottom left) or on cells in mid S-phase (bottom right). Note that focusing the analysis on specific sub-populations only (e.g. excluding cells in G1 and G2) enhances the dynamic range of the analysis. Color codes as defined in the figure panels. Scale bars, 10 µm.

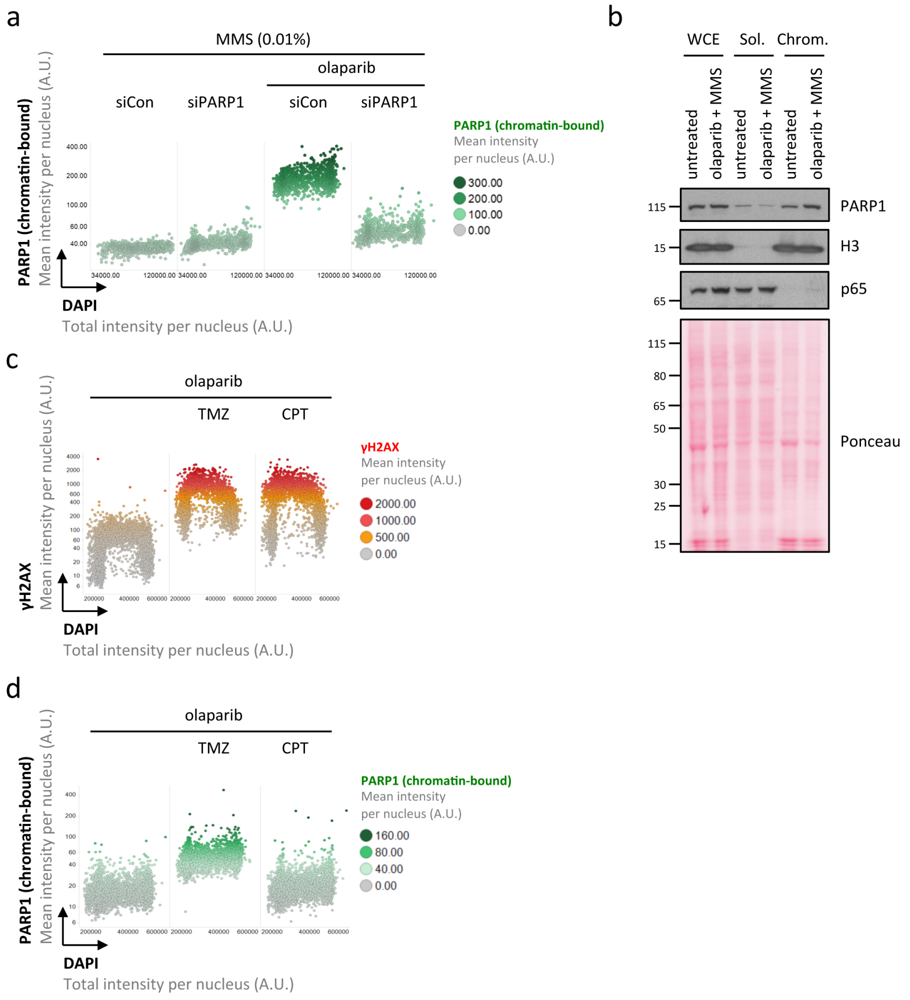

Supplementary Figure 4: Image-based quantification of PARPi-induced PARP trapping in conjunction with different genotoxic agents. (a) U-2 OS cells were transfected with siRNAs as indicated and treated with 10 µM olaparib and 0.01% MMS for 4 hours, pre-extracted on ice in 0.2% Triton X-100 for 2 min to remove soluble, non-chromatin-bound proteins, and stained for PARP1 and DNA content. Chromatin-bound PARP1 levels were quantified and are depicted as a function of cell cycle progression. **(b)** U-2 OS cells were treated with 10 µM olaparib and 0.01% MMS for 4 hours as indicated,and whole cell extracts (WCE), soluble (Sol.) and chromatin fractions (Chrom.) were analyzed for levels of PARP1. H3 and p65 served as loading and fractionation controls. **(c)** Cells were treated with 10 µM olaparib for 4 hours in the presence of 1 mM TMZ or 1 µM CPT, pre-extracted on ice in 0.2% Triton X-100 for 2 min to remove soluble, non-chromatin-bound proteins, and stained for PARP1, γH2AX and DNA content. Cell cycle resolved γH2AX profiles are shown. **(d)** For the same cell populations, cell cycle resolved PARP1 trapping is shown. Color codes as defined in the figure panels.

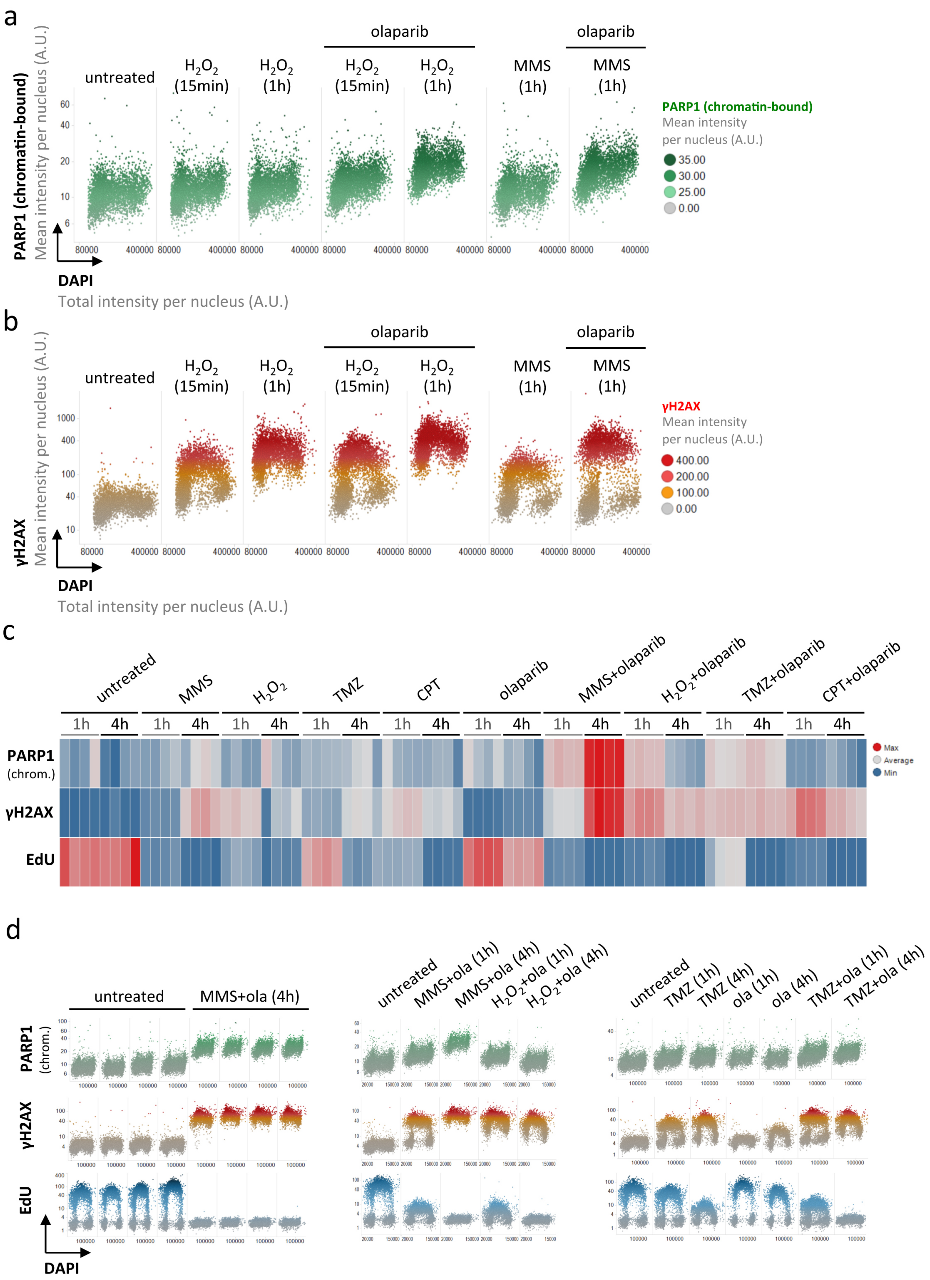

Supplementary Figure 5: Image-based quantification of PARPi-induced PARP trapping in conjunction with different genotoxic agents. (a) U-2 OS cells were treated with H_2O_2 (0.1 mM), olaparib (10 µM) and MMS (0.01%) as indicated, pre-extracted on ice in 0.2% Triton X-100 for 2 min to remove soluble, non-chromatin-bound proteins, and stained for PARP1, γH2AX and DNA content. Chromatin-bound PARP1 levels were quantified and are depicted as a function of cell cycle progression. **(b)** For the same cell populations, cell cycle resolved γH2AX profiles are shown. **(c)** Cells were grown on a multiwell plate, treated with MMS (0.01%), H_2O_2 (0.1 mM), olaparib (10 µM), TMZ (0.1 mM) or CPT (1 µM) as indicated for 1 or 4 hours, pulsed with EdU for the last 20 min, pre-extracted on ice in 0.2% Triton X-100 for 2 min to remove soluble, non-chromatin-bound proteins, and stained for EdU, γH2AX, PARP1 and DNA content. (**d**) Cell cycle resolved γH2AX, chromatin-bound PARP1 and EdU profiles for specific treatments obtained in (c) are shown. Color codes as defined in the figure panels.

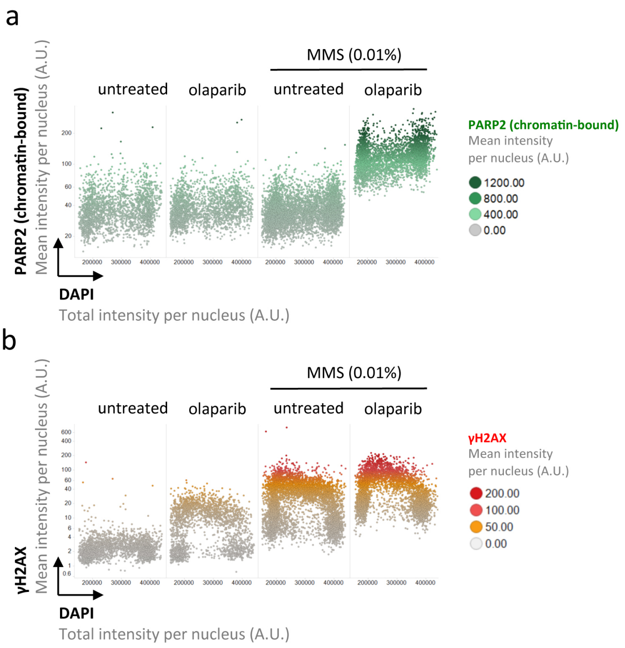

Supplementary Figure 6: Image-based quantification of PARPi-induced PARP2 trapping and DNA damage signaling. **(a)** Cells were treated with olaparib (10 µM) and MMS (0.01%) for 4 hours as indicated, pre-extracted on ice in 0.2% Triton X-100 for 2 min to remove soluble, non-chromatin-bound proteins, and stained for PARP2, γH2AX and DNA content. Chromatin-bound PARP2 levels were quantified and are depicted as a function of cell cycle progression. **(b)** For the same cell populations, γH2AX levels were quantified and are depicted as a function of cell cycle progression.

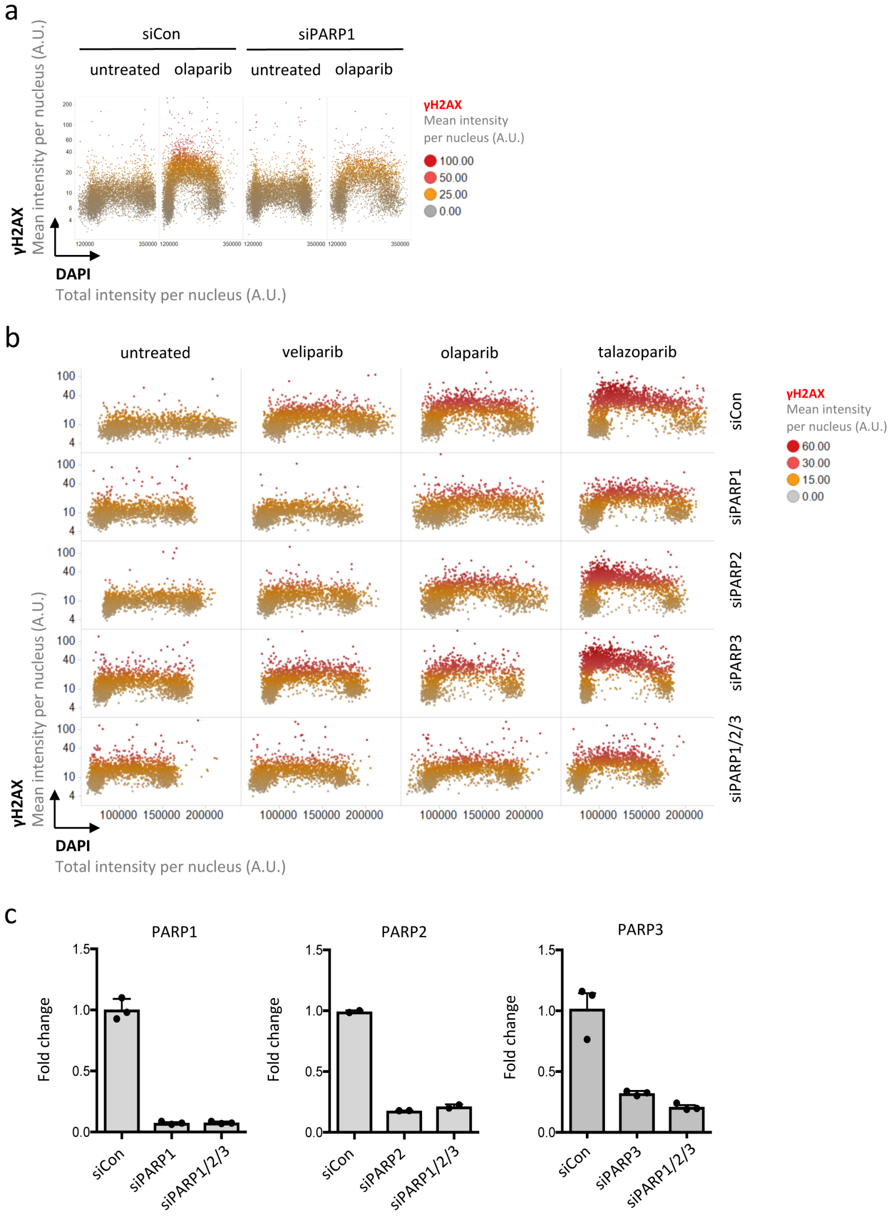

Supplementary Figure 7: Depletion of PARP1 and PARP2 alleviates PARPi toxicity. (a) Cells were transfected with siRNA against PARP1 and treated with 10 µM olaparib for 8 hours. Cell cycle resolved γH2AX profiles are shown. Color codes as defined in the figure panels. **(b)** Cells were transfected with siRNAs against PARP1, PARP2, and PARP3 as indicated and treated with olaparib, veliparib and talazoparib (all 10 µM) for 8 hours. Cell cycle resolved γH2AX profiles are shown. Color codes as defined in the figure panels. **(c)** qPCR analysis confirmed PARP1, PARP2 and PARP3 down-regulation in siRNA treated samples. Data are presented as fold-change (mean ± s.d.) relative to siCon-transfected samples.

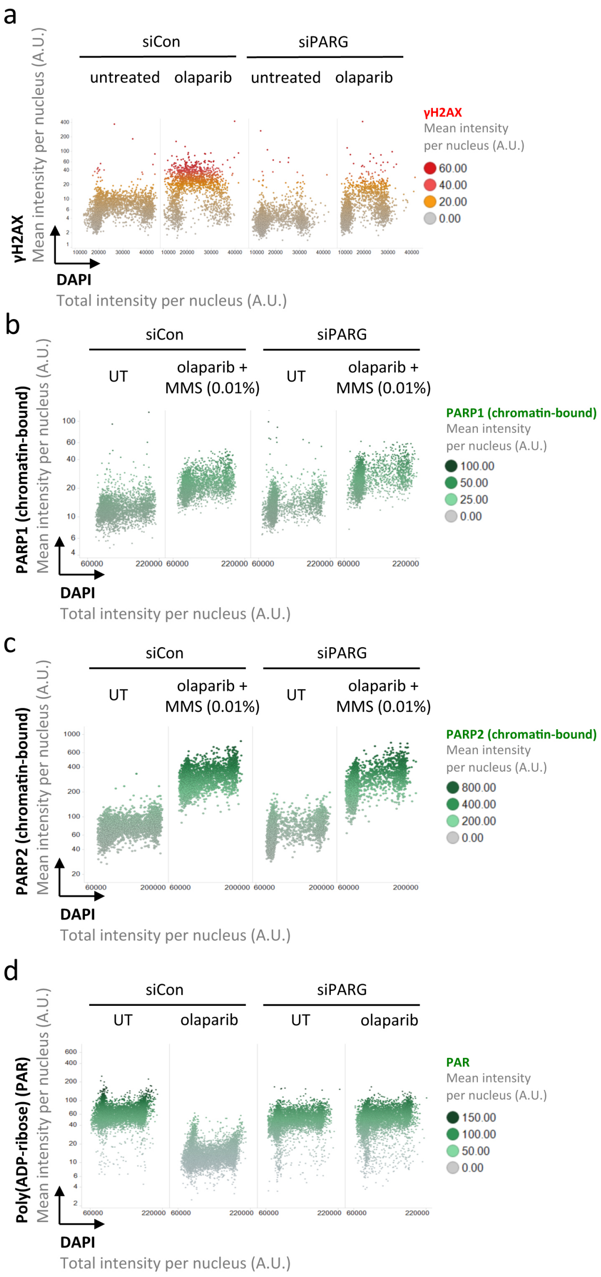

Supplementary Figure 8: Resistance to PARPi toxicity by down-regulation of PARG. (a) U-2 OS cells were transfected with siRNA against PARG, treated with 10 µM olaparib for 8 h, and stained for γH2AX and DNA content. Cell cycle resolved γH2AX profiles are shown. **(b)** Cells transfected with siRNA against PARG were treated with 10 µM olaparib and 0.01% MMS for 4 hours as indicated, pre-extracted on ice in 0.2% Triton X-100 for 2 min to remove soluble, non-chromatin-bound proteins, and stained for PARP1 and DNA content. **(c)** Cells were treated as in (b) and stained for PARP2 and DNA content. **(d)** Cells transfected with siRNA against PARG were treated with 10 µM olaparib for 1 hour and stained for PAR and DNA content. Cellular PAR levels were quantified and are depicted as a function of cell cycle progression. Color codes as defined in the figure panels.

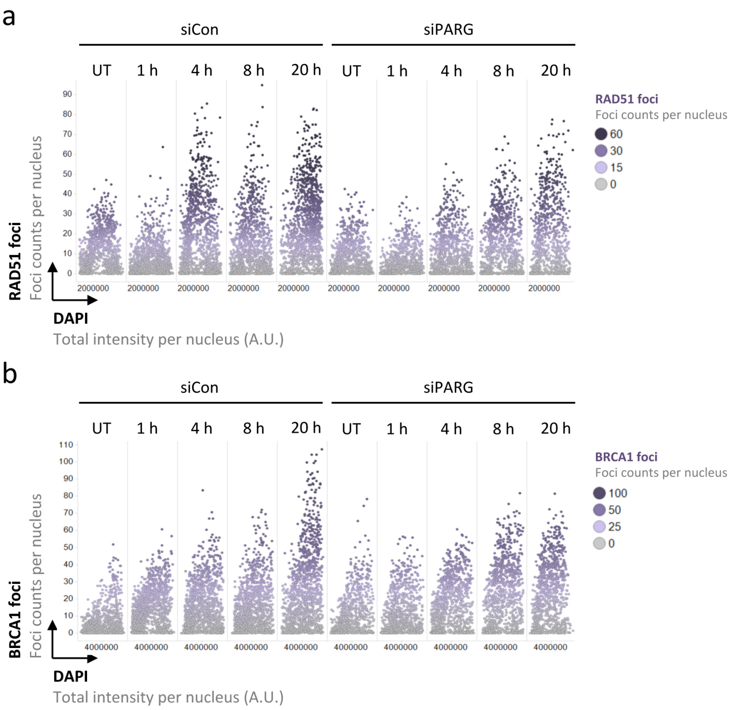

Supplementary Figure 9: Down-regulation of PARG alleviates RAD51 and BRCA1 foci formation upon PARPi. (a) U-2 OS cells were transfected with siRNA against PARG, treated with 10 µM olaparib for different time periods as indicated and stained for RAD51, BRCA1 and DNA content. RAD51 foci were quantified in large cohorts of asynchronously growing cells and RAD51 foci numbers per cell are depicted as a function of cell cycle progression. **(b)** For the same cell populations BRCA1 foci numbers per cell are depicted as a function of cell cycle. Color codes as defined in the figure panels.

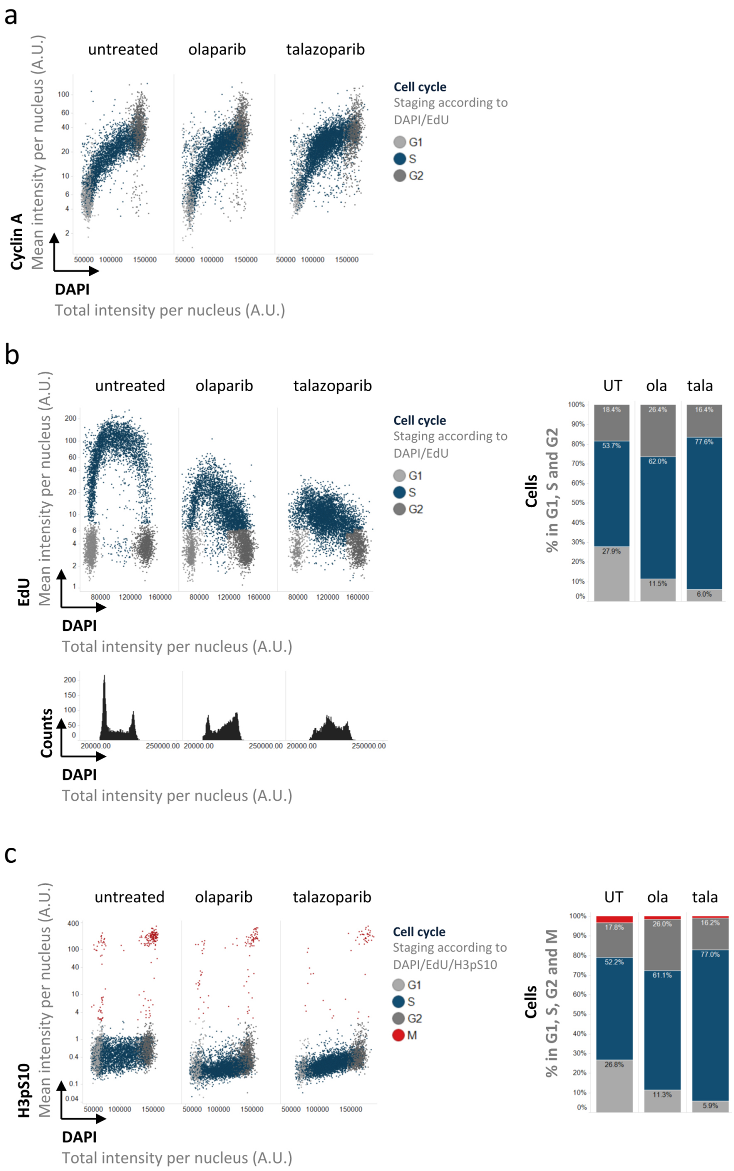

Supplementary Figure 10: PARPi-induced cell cycle arrest revealed by 4-dimensional quantitative image-based cytometry. (a) U-2 OS cells were treated with PARPi olaparib or talazoparib (both 10 µM) for 24 hours, pulsed with EdU for the last 20 min, and stained for EdU, Cyclin A, H3pS10 and DAPI. Two-dimensional cell cycle staging was performed based on the DAPI/Cyclin A profile. **(b)** For the same cell populations two-dimensional cell cycle staging based on the DAPI/EdU is shown. One-dimensional cell cycle profiles are shown below. Percentages of cells in G1, S and G2 are shown on the right. UT, untreated; ola, olaparib; tala, talazoparib. **(c)** For the same cell populations two-dimensional cell cycle staging was performed based on the DAPI/H3pS10 profile. Percentages of cells in G1, S, G2 and M are shown on the right. UT, untreated; ola, olaparib; tala, talazoparib. Color codes as defined in the figure panels.

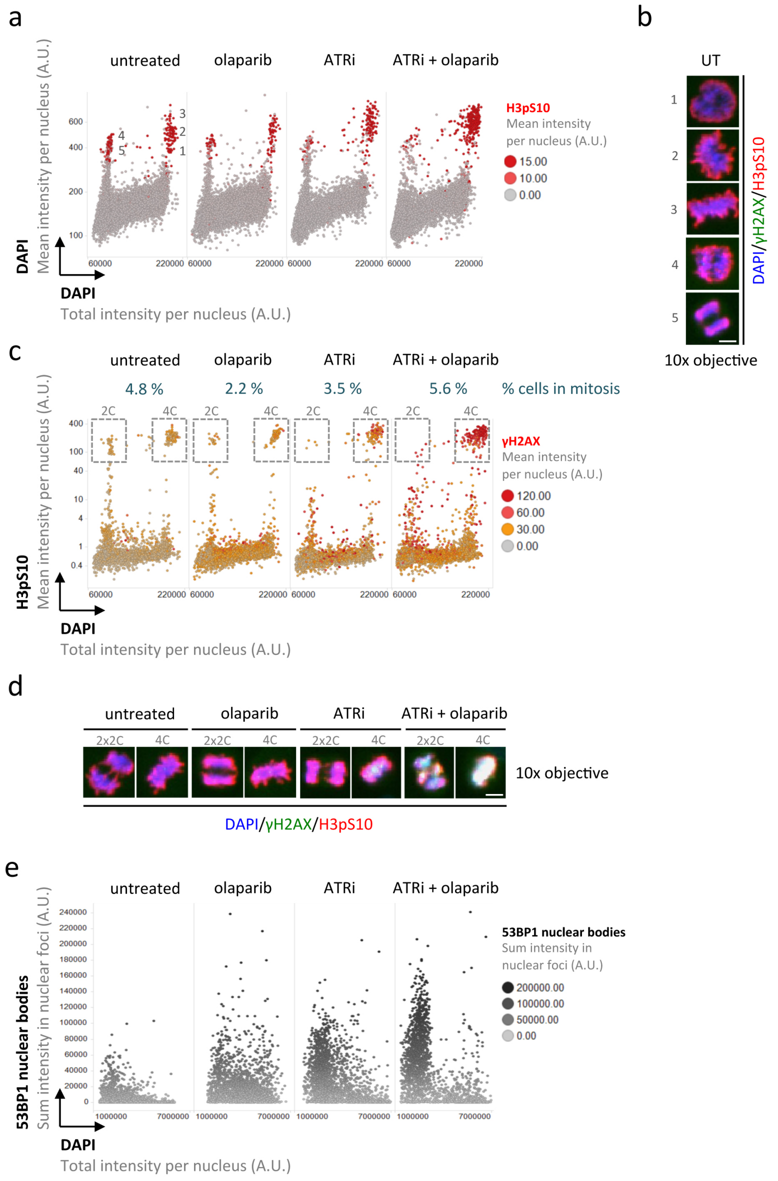

Supplementary Figure 11: Synthetic lethality between PARPi and ATRi involves transmission of replication-born lesions into mitosis. (a) U-2 OS cells were treated for 24h with olaparib (10 µM) and ATRi (1 µM) as indicated and stained for H3pS10, γH2AX and DAPI. Total versus mean DAPI intensities color-coded for H3pS10 are shown. Mitotic cells are characterized by increased mean DAPI intensity and high H3pS10. **(b)** Representative images of cells from (a) in different phases of mitosis (1-5) are shown. **(c)** For the same cell populations in (a), two-dimensional cell cycle staging was performed based on DAPI/H3pS10. Percentages indicate cells in mitosis upon the different treatments. **(d)** Representative images of mitotic cells in anaphase (2C) and metaphase (4C) after single and combined treatment. **(e)** Cells were treated for 24h with olaparib (10 µM) and ATRi (1 µM) as indicated and stained for 53BP1 and DNA content. 53BP1 nuclear bodies (measured as accumulated intensity of 53BP1 in sub-nuclear foci) are depicted as a function of cell cycle progression. Color codes as defined in the figure panels. Scale bars, 5 µm.

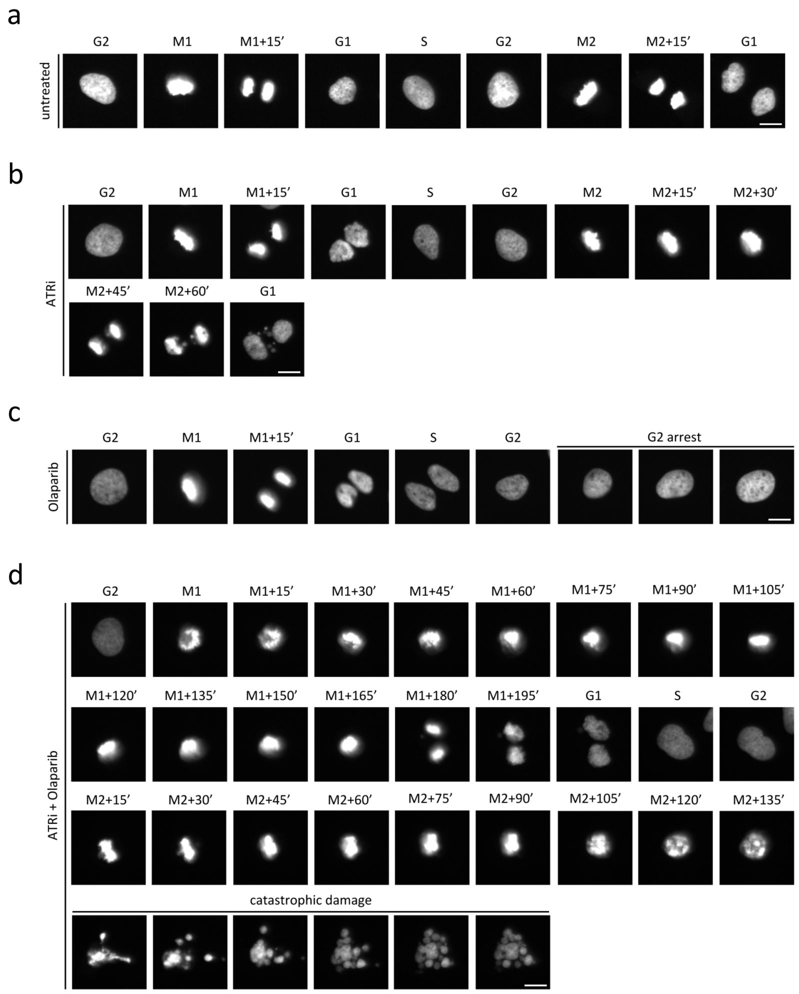

Supplementary Figure 12: Synthetic lethality between PARPi and ATRi involves transmission of replication-born lesions into mitosis and causes catastrophic damage. H2B-GFP U-2 OS cells were left untreated **(a)** or exposed to ATRi (1 µM) **(b)**, PARPi (10 µM) **(c)**, or a combination of both **(d)** and time-lapse microscopy was performed at 15-minute intervals for up to 48h. Progression through a first (M1) and second (M2) mitosis was followed. Scale bars, 10 µm.

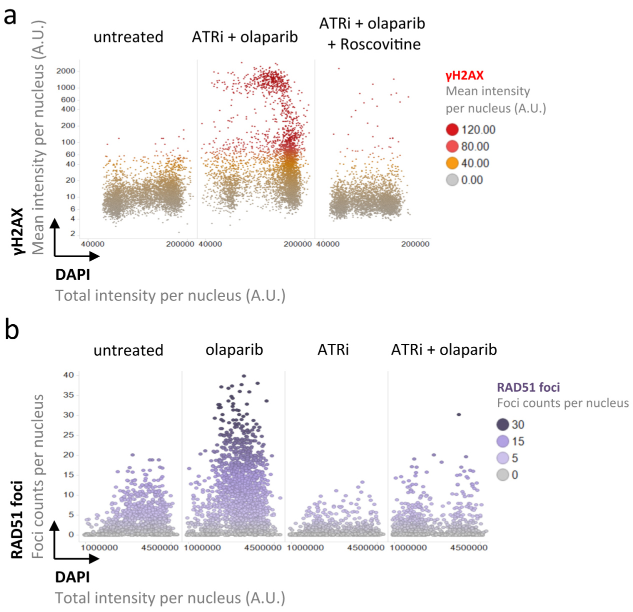

Supplementary Figure 13: Synthetic lethality between PARPi and ATRi. (a) U-2 OS cells were treated with a combination of PARPi and ATRi for 24h in the absence of presence of CDK inhibitor Roscovitine (20 µM). Cell cycle resolved γH2AX profiles are shown. **(b)** Cells were treated for 24h with olaparib (10 µM) and ATRi (1 µM) as indicated and stained for RAD51 and DNA content. RAD51 foci were quantified in large cohorts of asynchronously growing cells and RAD51 foci numbers per cell are depicted as a function of cell cycle progression. Color codes as defined in the figure panels.

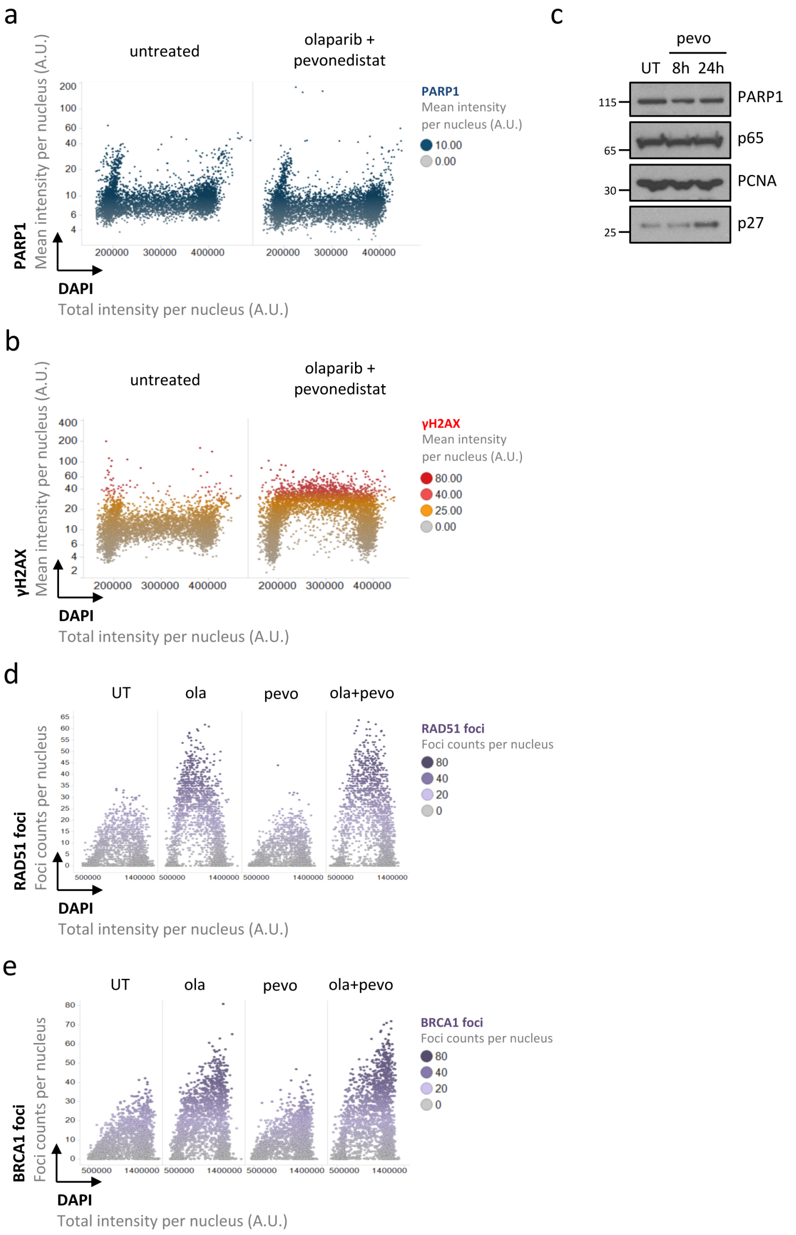

Supplementary Figure 14: A targeted screen identifies a functional interaction between the NEDD8/SCF machinery and PARP inhibition. (a) U-2 OS cells were treated with 10 µM olaparib and 100 nM pevonedistat for 8 hours and stained for PARP1, γH2AX and DNA content. Cell cycle resolved PARP1 profiles are shown. **(b)** For the same cell population cell cycle resolved γH2AX profiles are shown. **(c)** U-2 OS cells were treated with 100 nM pevonedistat for 8 and 24 hours and protein extracts were probed for PARP1, p65, PCNA and p27. Elevated levels of p27 upon prolonged pevonedistat treatment served as positive treatment control. Pevo, pevonedistat. **(d)** U-2 OS cells were treated with 10 µM olaparib and 100 nM pevonedistat for 8 hours and stained for RAD51, BRCA1 and DNA content. RAD51 foci were quantified in large cohorts of asynchronously growing cells and RAD51 foci numbers per cell are depicted as a function of cell cycle progression. UT, untreated; ola, olaparib; pevo, pevonedistat. **(e)** For the same cell population BRCA1 foci numbers per cell are depicted as a function of cell cycle. Color codes as defined in the figure panels. UT, untreated; ola, olaparib; pevo, pevonedistat.

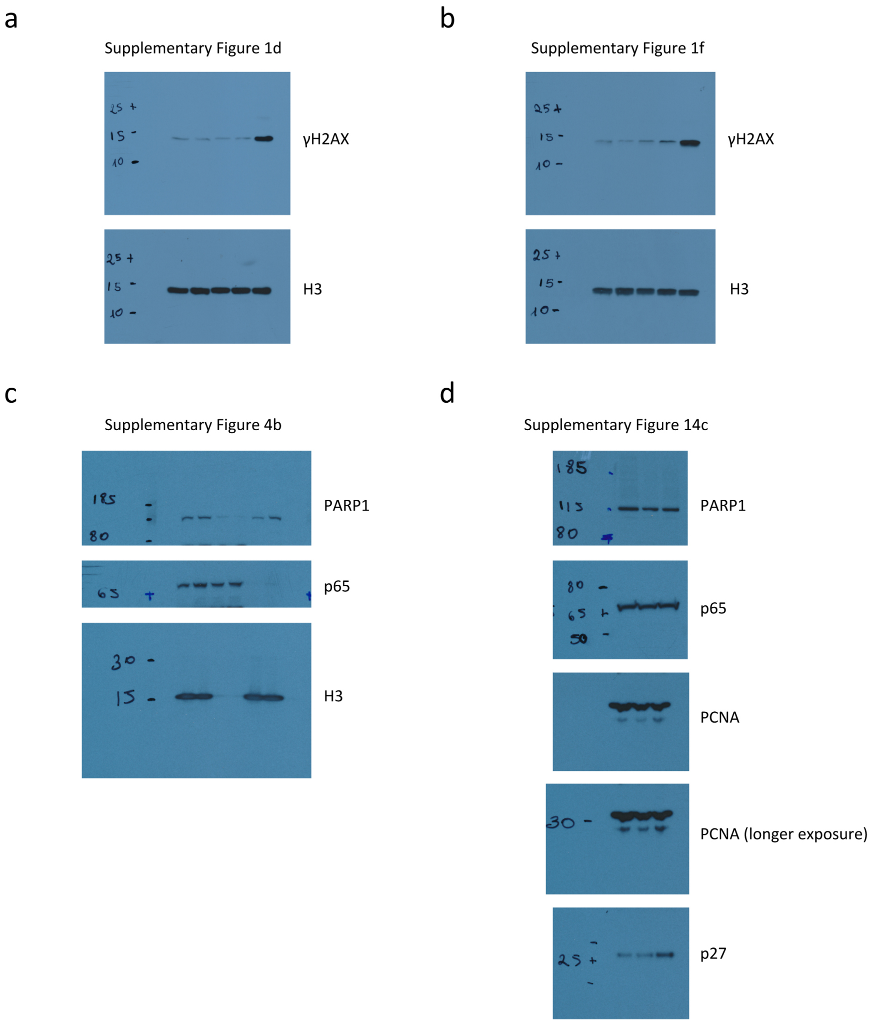

Supplementary Figure 15: Scans of Western Blots. (a) Scanned Western Blot films used to assemble Supplementary Figure 1d. **(b)** Scanned Western Blot films used to assemble Supplementary Figure 1f. **(c)** Scanned Western Blot films used to assemble Supplementary Figure 4b. **(d)** Scanned Western Blot films used to assemble Supplementary Figure 14c. Molecular weight markers are indicated.
